# Transcriptional Regulation of BMP2 Expression by the PTH-CREB Signaling Pathway in Osteoblasts

**DOI:** 10.1371/journal.pone.0020780

**Published:** 2011-06-09

**Authors:** Rongrong Zhang, James R. Edwards, Seon-Yle Ko, Shanshan Dong, Hongbin Liu, Babatunde O. Oyajobi, Christopher Papasian, Hong-Wen Deng, Ming Zhao

**Affiliations:** 1 Department of Biostatistics and Bioinformatics, Tulane University, New Orleans, Louisiana, United States of America; 2 Department of Medicine, Vanderbilt University, Nashville, Tennessee, United States of America; 3 School of Dentistry, Dankook University, Cheonan, Choongnam, Korea; 4 Department of Cellular and Structural Biology, University of Texas Health Science Center at San Antonio, San Antonio, Texas, United States of America; 5 Department of Basic Medical Sciences, University of Missouri – Kansas City, Kansas City, Missouri, United States of America; 6 Department of Cellular and Molecular Biology, Tulane University, New Orleans, Louisiana, United States of America; Clermont Université, France

## Abstract

Intermittent application of parathyroid hormone (PTH) has well established anabolic effects on bone mass in rodents and humans. Although transcriptional mechanisms responsible for these effects are not fully understood, it is recognized that transcriptional factor cAMP response element binding protein (CREB) mediates PTH signaling in osteoblasts, and that there is a communication between the PTH-CREB pathway and the BMP2 signaling pathway, which is important for osteoblast differentiation and bone formations. These findings, in conjunction with putative cAMP response elements (CREs) in the BMP2 promoter, led us to hypothesize that the PTH-CREB pathway could be a positive regulator of BMP2 transcription in osteoblasts. To test this hypothesis, we first demonstrated that PTH signaling activated CREB by phosphorylation in osteoblasts, and that both PTH and CREB were capable of promoting osteoblastic differentiation of primary mouse osteoblast cells and multiple rodent osteoblast cell lines. Importantly, we found that the PTH-CREB signaling pathway functioned as an effective activator of BMP2 expression, as pharmacologic and genetic modulation of PTH-CREB activity significantly affected BMP2 expression levels in these cells. Lastly, through multiple promoter assays, including promoter reporter deletion, mutation, chromatin immunoprecipitation (ChIP), and electrophoretic mobility shift assay (EMSA), we identified a specific CRE in the BMP2 promoter which is responsible for CREB transactivation of the BMP2 gene in osteoblasts. Together, these results demonstrate that the anabolic function of PTH signaling in bone is mediated, at least in part, by CREB transactivation of BMP2 expression in osteoblasts.

## Introduction

Parathyroid hormone (PTH) plays an important role in skeletal metabolism. In mice [1]–[4] and rats [5], [6], intermittent administration of PTH has anabolic effects on bone mass and bone formation. Similarly, when PTH is administered intermittently to humans, it has been proven to increase bone mass and decrease fracture risk in post-menopausal women, elderly men, and women with glucocorticoid-induced osteoporosis [7]–[10]. Despite its clinical efficacy, the precise molecular mechanisms responsible for the anabolic effects of PTH on bone, particularly at the transcriptional level, need to be further elucidated.

Although multiple intracellular signaling mechanisms are involved in mediating PTH function, the major downstream signaling pathway of PTH in bone cells involves 5′-cyclic adenosine monophosphate (cAMP), protein kinase A (PKA), and cAMP response element binding protein (CREB). The anabolic function of this cAMP-PKA-CREB pathway in bone has been characterized *in vivo* and *in vitro* [11]–[30]. Increasing activity of this pathway promotes osteoblast differentiation [11]–[26] and stimulates bone formation [27]–[30]. In osteoblasts, PTH binds to a PTH receptor and induces formation of cAMP, leading to activation of PKA, which in turn phosphorylates and activates CREB, a member of a large family of basic leucine zipper (bZIP) domain DNA-binding proteins [11]–[14]. Activated CREB, in association with the other co-activators, binds to target genes through a cAMP response element (CRE) and activates their transcription. Therefore, in this pathway, CREB plays a pivotal role by converting the PTH signal to activation of gene expression. In various cell systems, CREB induces the expression of some osteoblast-related genes, such as bone sialoprotein (BSP) and osteocalcin (OCN), by directly binding to these promoters [13]–[22]. However, the major downstream targets of CREB transactivation, which have a predominant role in initiating osteoblast differentiation and stimulating bone formation, are unknown.

Bone morphogenetic protein 2 (BMP2) is an important growth factor that stimulates osteoblast differentiation and bone formation [31]–[33]. The PTH-cAMP-CREB signaling pathway synergizes the anabolic signaling of BMP2 for osteoblast differentiation and bone formation [23], [25], [26], [28], [29], [34], indicating that there is a communication between the PTH-cAMP-CREB and BMP2 signaling pathways. Recently, we have reported that osteoblast-specific deficiency of CREB in mice causes reduction of postnatal bone mass and decreases BMP2 expression in osteoblasts [35], [36], suggesting that BMP2 could be a critical transcriptional target of CREB in osteoblasts. This is consistent with the findings of a genome-wide study, based on a chromatin immunoprecipitation (ChIP) assay, in which the cAMP-CREB signaling pathway was identified as a positive transcriptional regulator of BMP2 mRNA expression in PC12 cells [37]. Furthermore, we have identified multiple CREs in the BMP2 promoter by DNA sequence analysis. Based on the collective findings reviewed above, it is reasonable to hypothesize that the transcription factor CREB is an activator of BMP2 expression in osteoblasts, and that CREB mediates the anabolic function of PTH-cAMP signaling in bone, at least in part, by this mechanism. This hypothesis was examined in the current investigation through pharmacological and genetic experiments, and promoter analyses.

## Results

### PTH signaling activates CREB in osteoblasts

**Figure 1:**
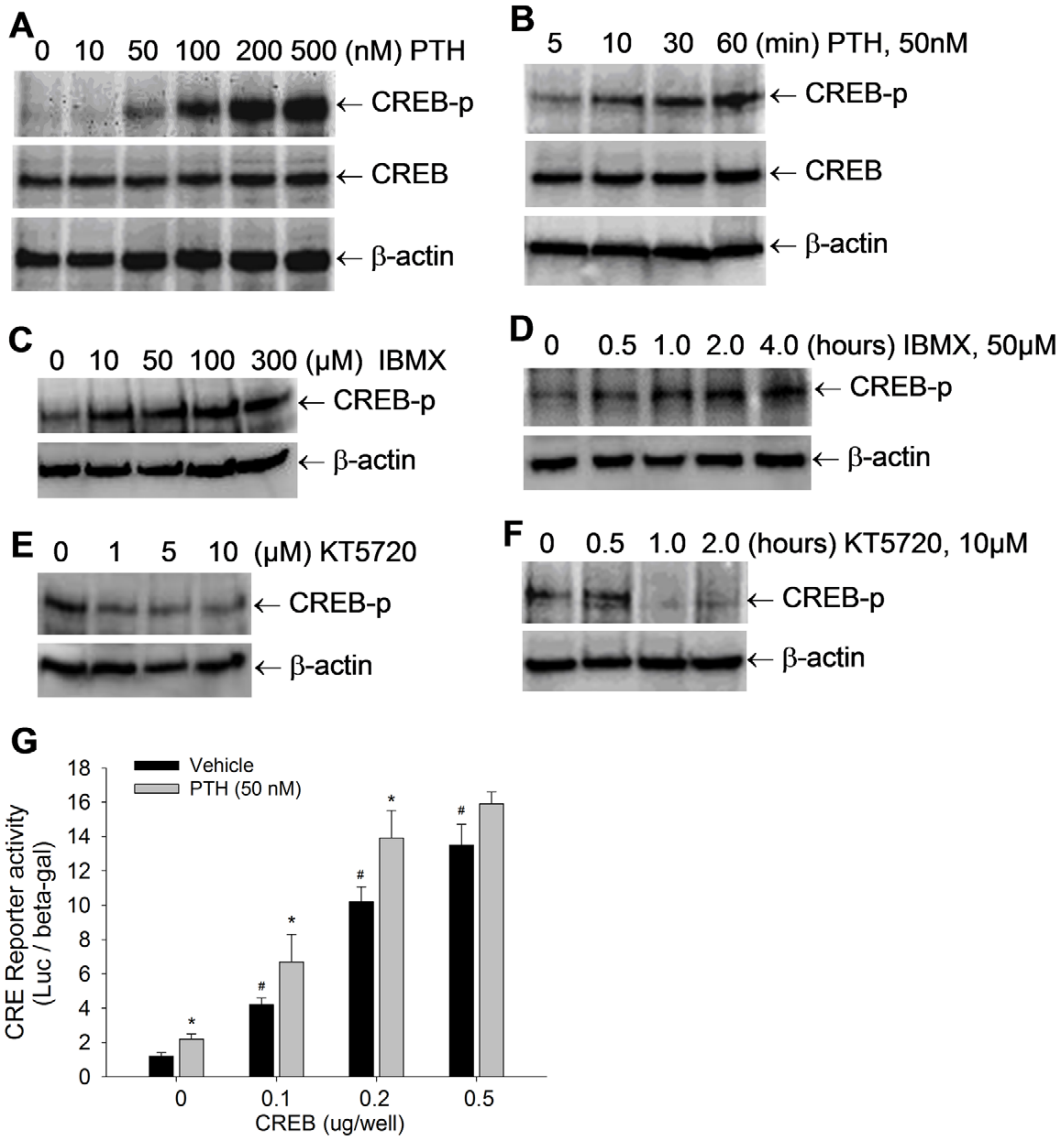
PTH signaling activates CREB phosphorylation in osteoblasts. (A–F) CREB phosphorylation levels in C2C12 cells, treated with PTH at 0 to 500 nM for 1 hour (A); PTH at 50 nM for 5 to 60 min (B); or IBMX at 0 to 300 µM for 1 hour (C); IBMX at 50 µM for 0 to 4 hours (D); or KT5720 at 0 to 10 µM for 1 hour (E); KT5720 at 10 µM for 0 to 2 hours (F), were detected by Western blot with anti-phosphorylated CREB antibody, with normalization by non-phosphorylated CREB and β-actin. (G) C2C12 cells were co-transfected with CRE-Luc reporter and CREB expression vector, and treated with PTH at 50 nM for 36 hours. The reporter luciferase activity was measured with normalization by β-gal activity. # p<0.01 (CREB vs vector; mean±SE, n = 6); * p<0.05 (PTH vs vehicle; mean±SE, n = 6).

CREB activity is controlled by phosphorylation through multiple signaling pathways, including the PTH signaling pathway [11]–[14]. Here, we determined the effects of manipulating the signaling activity of the PTH-cAMP-signaling cascade on CREB phosphorylation in osteoblasts. First, we determined the effect of PTH on CREB phosphorylation in osteoblasts. Pluripotent mesenchymal precursor C2C12 cells, which are capable of differentiating into an osteoblast lineage, were treated with PTH, and phosphorylation levels of CREB were detected by Western blot, with non-phosphorylated CREB and β-actin as controls. The results showed that treatment with PTH at 0–500 nM substantially, and dose-dependently, increased CREB phosphorylation (Fig. 1A). The minimum dose with a discernable effect was approximately 50 nM (Fig. 1A). The PTH-induced CREB phosphorylation in osteoblasts was also time-dependent between 5 and 60 minutes (Fig. 1B). As a second messenger, cAMP is known to mediate PTH signaling. Thus, we determined the effects of the cAMP enhancer 3-isobutyl-1-methylxanthine (IBMX), an inhibitor of cAMP phosphodiesterase, on CREB phosphorylation. Western blot showed that incubation of C2C12 cells with IBMX at doses of 10–300 µM increased intracellular levels of phosphorylated CREB protein (Fig. 1C), and that this stimulation was induced in a time-dependent manner up to 4 hours (Fig. 1D). As a direct downstream target of cAMP, PKA phosphorylates CREB. Next, we examined the effects of inhibition of PKA activity on CREB phosphorylation using KT5720, a PKA inhibitor. We found that the KT5720-induced inhibition of PKA activity markedly reduced levels of phosphorylated CREB in C2C12 cells, in both dose- and time-dependent manners (Fig. 1E, 1F). The levels in the Western blot of phosphor-CREB treated by IBMX and KT5720 were quantitated. The quantitative results confirmed that these cAMP and PKA drugs dose- and time-dependently regulated CREB phosphorylation in osteoblasts (see the quantitative data in Figure S1).

Phosphorylated CREB migrates into nuclei and transactivates target genes through CRE binding sites. Consequently, we evaluated the effects of PTH, which induces CREB phosphorylation, on transcriptional activity of CREB. The luciferase assay using a CREB-specific reporter CRE-Luc showed that overexpression of CREB in C2C12 cells dose-dependently increased the activity of the CRE reporter, and that treatment with PTH at 50 nM further elevated the CREB-induced reporter activity (Fig. 1G).

### PTH-CREB pathway promotes osteoblast differentiation

**Figure 2:**
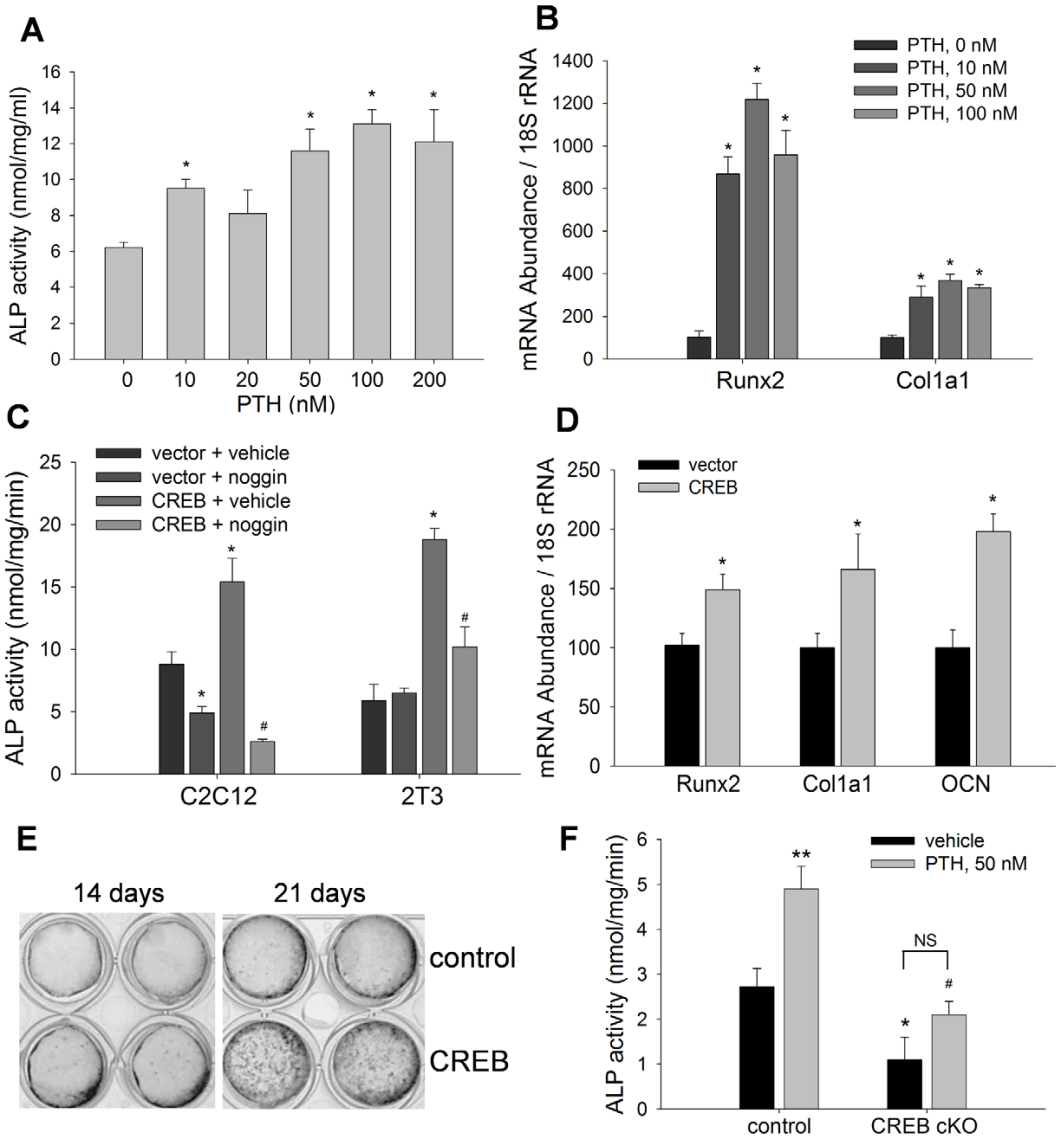
PTH-CREB pathway promotes osteoblast differentiation. (A) ALP activity in C2C12 cells, treated with PTH at 0 to 200 nM for 24 hours, was measured with normalization by cell proteins. * p<0.05 (vs vehicle; mean±SE, n = 8). (B) mRNA levels of Runx2 and Col1a1 in C2C12 cells, treated with PTH for 12 hours, were quantitated by real time PCR, normalized by 18S rRNA. * p<0.01 (vs vehicle; mean±SE, n = 6). (C) ALP activity in C2C12 and 2T3 cells was measured after transfection with CREB expression plasmid and treated with noggin at 500 nm/ml for 48 hours. * p<0.01 (CREB vs vector); # p<0.05 (noggin vs vehicle; mean±SE, n = 8). (D) mRNA levels of Runx2, Col1a1 and OCN in C2C12 cells, transfected with CREB for 24 hours, were measured by real time PCR, normalized by 18S rRNA. * p<0.05 (vs vector; mean±SE, n = 6). (E) 2T3 cells were transfected with vector (top) or CREB plasmid (bottom) and cultured under osteogenic conditions for 14 and 21 days. Von Kossa staining was performed to visualize the mineralized matrix formation. (F) Isolated calvarial osteoblastic cells from newborn conditional CREB knockout mice (CREB cKO) and their CREBfloxed littermate controls were cultured for 24 hours and ALP activity was measured. * p<0.05 (cKO vs control); # p<0.05 (PTH on cKO vs PTH on control); ** p<0.05 (PTH va vehicle on control mice; mean±SE, n = 6); NS: not significant.

It has been postulated that the anabolic effect of PTH on bone is mediated through osteoblasts. In this series of experiments, we characterized the role of the PTH-CREB signaling pathway in osteoblast differentiation. First, we determined the effects of PTH on alkaline phosphatase (ALP) activity of C2C12 cells by incubating cells with PTH for 24 hours. Measurement of ALP activity in cell lysates showed that treatment with PTH at 0–200 nM stimulated ALP activity in a pattern that approached dose-dependence, with a maximum effect (2-fold increase) at PTH concentration 100 nM (Fig. 2A). Real time PCR showed that the mRNA levels of osteoblast maturation genes, Runx2 and type I collagen 1a1 (Col1a1), were significantly enhanced by treatment with PTH at doses of 10–100 nM (Fig. 2B).

Then, we determined the effects of overexpression of CREB on osteoblast differentiation. Osteoblast precursor C2C12 and 2T3 cells [38]–[40] were transfected with CREB expression plasmid or its empty vector for 48 hours. Enzymatic quantitation of ALP activity showed that CREB overexpression significantly increased ALP activity in both C2C12 and 2T3 cells, compared with vehicle controls (Fig. 2C). Interestingly, CREB-stimulation of ALP activity was completely reversed in these cells by adding noggin (500 ng/ml), a natural BMP antagonist, to the culture medium (Fig. 2C). This finding is critical evidence for our hypothesis that the stimulatory effect of CREB on ALP depends on BMP signaling. mRNA measurement of C2C12 cells also showed that CREB transfection significantly increased expression of osteoblast-specific marker genes, including Runx2, Col1a1 and osteocalcin (OCN) (Fig. 2D). Next, we determined whether overexpression of CREB affects bone matrix formation in osteoblast precursor 2T3 cells, which can form mineralized matrices in culture under osteogenic conditions [33], [37]–[40]. Von Kossa staining showed that mineralized bone nodule formation was greatly enhanced in 2T3 cell cultures 14 and 21 days after CREB transfection, compared with control 2T3 cells transfected with empty vector (Fig. 2E).

In order to confirm the role of CREB in promoting osteoblast differentiation, we performed a loss-of-function study with primary CREB-deficient calvarial osteoblast cells. These cells were isolated from conditional CREB knockout mice (CREB cKO), that were generated by crossing CREB floxed mice with Col1a1-Cre mice. We have recently reported that these osteoblast-specific CREB knockout mice exhibit low bone mass [35], [36]. In the present study, we found that CREB deficiency markedly decreased both basal and PTH-induced ALP activity in calvarial osteoblast cells, compared with floxed control cells (Fig. 2F). We also noticed that, despite at a much lower level than PTH treatment in the control group, PTH appeared still capable of increasing ALP activity in the absence of CREB, compared with vehicle treatment. However, the statistical analysis did not show a significant difference between the treatment groups in the CREB deficient cells. This suggests that PTH stimulation of ALP activity was attenuated by removal of the CREB gene in the calvairal osteoblast cells, (Fig. 2F). Together, these results suggest that the PTH-CREB pathway is an activator of osteoblast differentiation.

### PTH up-regulates BMP2 expression through CREB in osteoblasts

**Figure 3:**
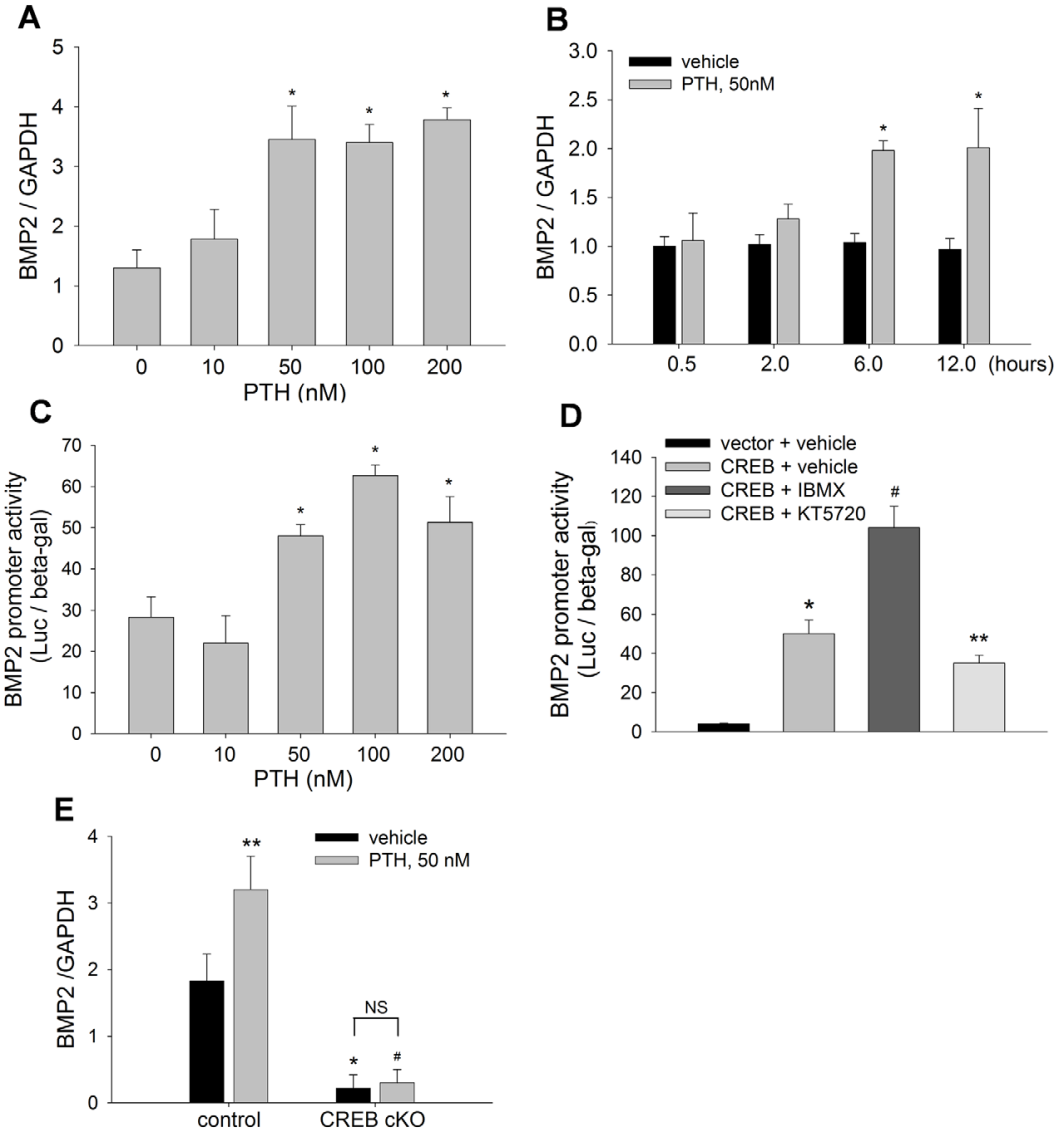
PTH up-regulates BMP2 expression through CREB. (A, B) BMP2 mRNA levels in C2C12 cells, treated with PTH at 0 to 200 nM for 12 hours (A); or PTH at 50 nM for 0 to 12 hours (B), were measured by real time PCR with GAPDH normalization. * p<0.01 (vs vehicle; mean±SE, n = 6). (C) BMP2 promoter reporter activity in C2C12 cells, transfected with −2712/+165-Luc and treated with PTH at 0 to 200 nM for 24 hours, was measured with β-gal normalization. * p<0.01 (vs vehicle; mean±SE, n = 8). (D) BMP2 promoter reporter activity in C2C12 cells, co-transfected with CREB plasmid or vector and reporter −2712/+165-Luc, and treated with IBMX at 50 µM or KT5720 at 5 µM for 24 hours, was measured. * p<0.01 (CREB vs vector); # p<0.01 (CREB+IBMX vs CREB+vehicle); ** p<0.05 (CREB+KT5720 vs CREB+vehicle; mean±SE, n = 8). (E) BMP2 mRNA levels in calvrial cells, isolated from CREB cKO and control mice, and treated with PTH at 50 nM for 24 hours, were measured by real time PCR. * p<0.01 (cKO vs control); # p<0.01 (PTH on cKO vs PTH on control); ** p<0.05 (PTH vs vehicle on control mice; mean±SE, n = 6); NS: not significant.

The finding that noggin blocked CREB-induced ALP activity in osteoblasts (Fig. 2C) suggested an involvement of the BMP pathway in CREB function. A previous genome-wide study has found that BMP2, a prototype member of the BMP family, is a transcriptional target of the CREB pathway in other cell systems [37]. Given these facts, and the presence of potential CREB binding elements in the BMP2 promoter (*vide infra*), we hypothesized that PTH-CREB signaling may function in bone, at least in part, by up-regulating BMP2 transcription in osteoblasts. To test this hypothesis, we first examined the effects of PTH on BMP2 expression. C2C12 cells were incubated with PTH at 0–200 nM for 12 hours, and real time PCR was performed to quantitate BMP2 mRNA levels. The results showed that PTH treatment increased BMP2 mRNA expression in a dose-dependent manner (Fig. 3A). We also found that stimulation of BMP2 mRNA expression by PTH (50 nM) was time-dependent from 0 to 12 hours (Fig. 3B). In BMP2 promoter assays, C2C12 cells were transfected with a mouse BMP2 promoter reporter, −2712/+165-Luc [38]–[40], and cultured in the presence or absence of PTH. The promoter reporter luciferase assays showed that treatment with PTH for 24 hours increased BMP2 promoter activity (Fig. 3C), further demonstrating that PTH is an activator of BMP2 transcription in osteoblasts.

Next, we determined the effects of drugs, IBMX and KT5720 that regulate PTH signaling activity, on BMP2 promoter activity. C2C12 cells that carry the BMP2 promoter reporter were transfected with CREB expression vector, and then treated with these drugs. We found that CREB transfection substantially increased BMP2 promoter activity, and that treatment with the cAMP enhancer IBMX further increased CREB stimulation. In contrast, treatment with the PKA inhibitor KT5720 significantly decreased CREB-induced BMP2 promoter activity (Fig. 3D).

Furthermore, we examined the effects of PTH on BMP2 transcription in the absence of CREB using the CREB-deficient calvarial cells described above. We found that CREB deficiency significantly reduced BMP2 mRNA levels compared with the control cells (Fig. 3E, black bars) and that the stimulation of BMP2 expression by treatment with PTH (50 nM) for 24 hours was largely abolished by inactivation of CREB in the CREB-deficient cells, compared with the control cells (Fig. 3E, gray bars). These data indicate that PTH signaling acts as an upstream stimulator of BMP2 gene expression in osteoblasts, and that this function is mediated through CREB.

### CREB activates BMP2 transcription in osteoblasts

**Figure 4:**
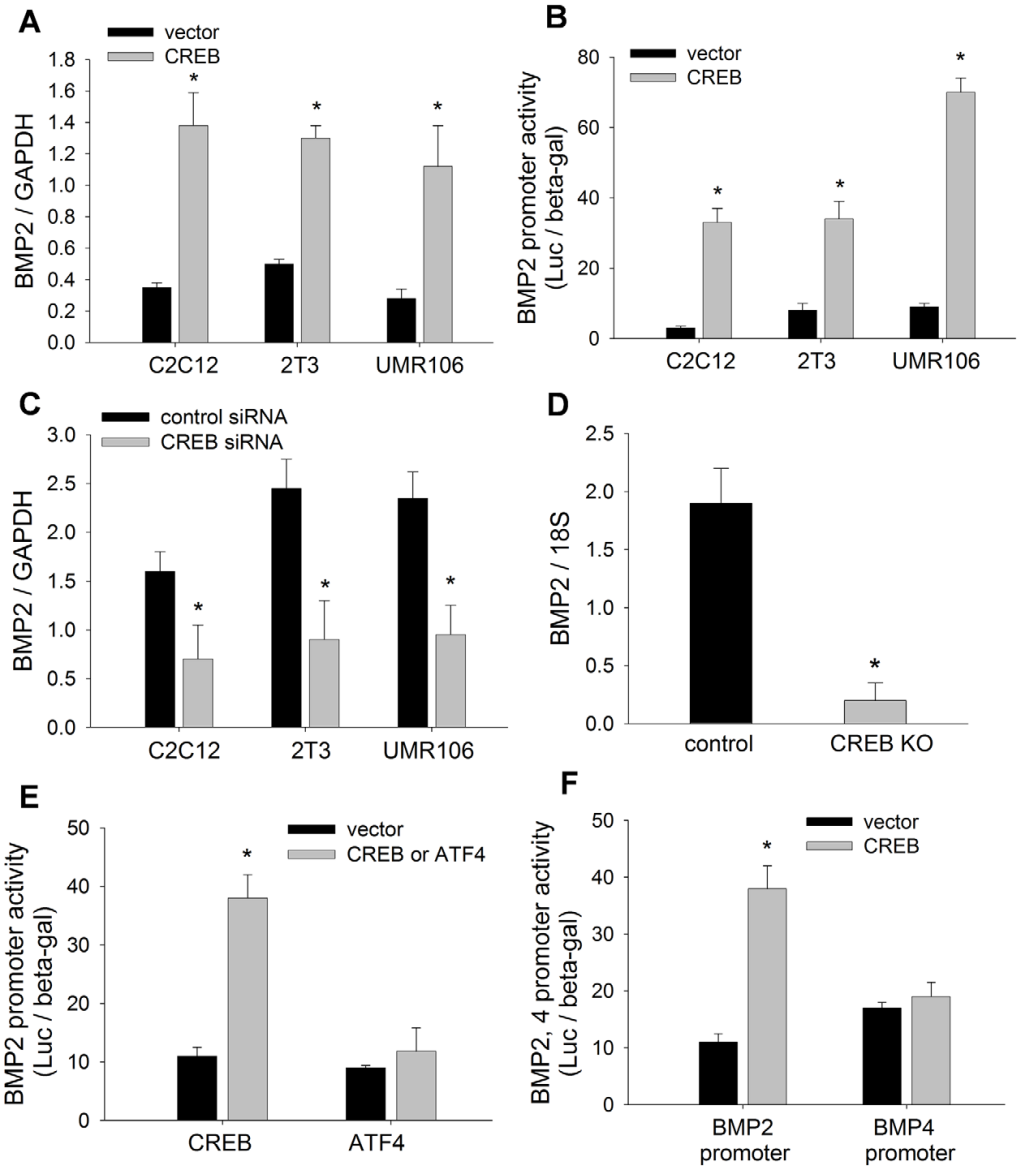
CREB activates BMP2 expression. (A) BMP2 mRNA levels in C2C12, 2T3 and UMR106 cells, transfected with CREB plasmid or vector for 24 hours, were measured by real time PCR with GAPDH106 cells. * p<0.01 (vs vector; mean±SE, n = 6). (B) BMP2 promoter activity in C2C12, 2T3 and UMR106 cells, co-transfected with −2712/+165-Luc and CREB plasmid for 24 hours, was measured by luciferase activity with β-gal normalization. * p<0.01 (vs vector; mean±SE, n = 8). (C) BMP2 mRNA levels in C2C12, 2T3 and UMR106 cells, transfected with CREB siRNA or control siRNA for 36 hours, were measured by real time PCR. * p<0.05 (vs control siRNA; mean±SE, n = 6). (D) BMP2 mRNA levels in calvarial cells of CREB cKO and control mice were measured by real time PCR. * p<0.01 (vs control; mean±SE, n = 6). (E) BMP2 promoter reporter activity in C2C12 cells, co-transfected with −2712/+165-Luc and CREB or ATF4 plasmid for 24 hours, was measured. * p<0.01 (vs vector; mean±SE, n = 8). (F) BMP2 promoter reporter activity in C2C12 cells, co-transfected with −2712/+165-Luc or BMP4-Luc and CREB plasmid for 24 hours, was measured. * p<0.01 (vs vector; mean±SE, n = 8).

We have shown that overexpression of CREB enhanced BMP2 promoter activity (Fig. 3D). To fully characterize the activating function of CREB on the BMP2 gene, we performed multiple gain- and loss-of-function experiments to determine the effects of manipulating CREB levels on BMP2 gene expression in osteoblasts. We found that overexpression of CREB in osteoblastic cell lines, including C2C12, 2T3 and UMR106 cells, substantially increased BMP2 mRNA levels in these cells, compared with vector controls (Fig. 4A). Next, we evaluated the effects of CREB transfection on BMP2 promoter activity. The luciferase assay with −2712/+165-Luc reporter showed that CREB markedly increased transcriptional activity of the BMP2 promoter in these osteoblastic cells, with a maximal stimulation of approximately 15-fold in C2C12 cells (Fig. 4B). In contrast, small interfering RNA (siRNA) knockdown of endogenous CREB in these osteoblast cell lines had significant inhibitory effects on BMP2 mRNA transcription, measured by real time PCR, compared with siRNA controls (Fig. 4C). The loss-of-function by siRNA was further confirmed by CREB knockout. mRNA quantitation demonstrated that an osteoblast-specific CREB deletion markedly reduced BMP2 mRNA levels in primary calvarial osteoblastic cells, compared with cells isolated from control mice (Fig. 4D).

In addition to CREB and BMP2, other members of the CREB and BMP families also are involved in osteoblast function. The closest members in these respective families are activating transcription factor 4 (ATF4) and BMP4. In order to test the specificity of the effects of CREB on BMP2 expression, we examined the effects of ATF4 on BMP2 expression and the effects of CREB on BMP4 expression. The factor ATF4 belongs to the same large bZIP family of transcription factors as CREB, and has proven to be important for osteoblast differentiation [41], [42]. We found that, compared with CREB stimulation, ATF4 transfection did not affect BMP2 promoter activity in C2C12 cells (Fig. 4E). In the BMP family, BMP4 is structurally closest to BMP2. Using a BMP4 promoter reporter, BMP4-Luc, we found that, CREB failed to enhance BMP4 promoter activity, in contrast to CREB enhancement of BMP2 promoter activity (Fig. 4F). Collectively, these results strongly support the conclusion that the transcriptional factor CREB is a specific and potent enhancer of BMP2 gene expression in osteoblasts.

### CREB transactivates BMP2 through CRE in the BMP2 promoter

**Figure 5:**
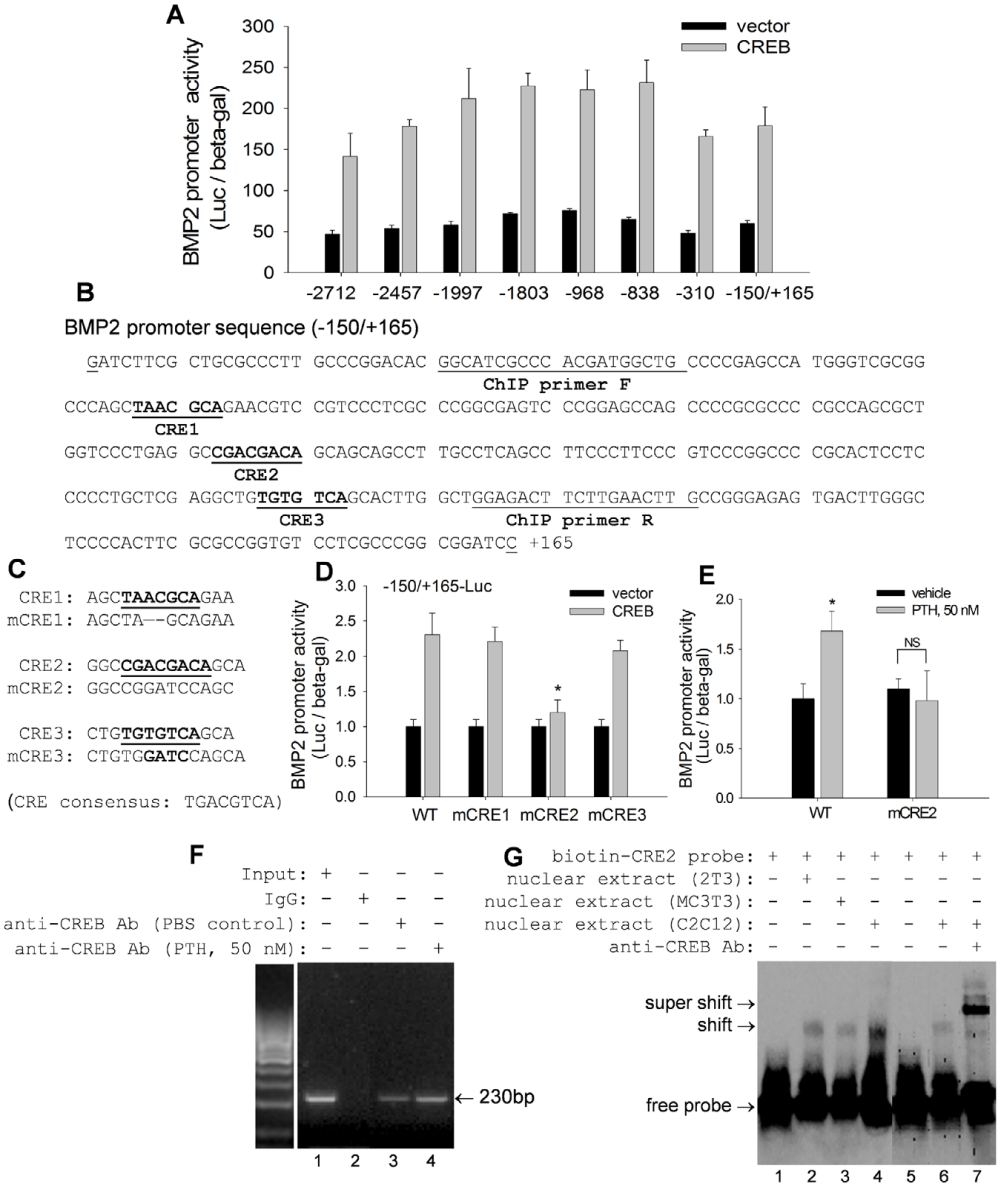
CREB transactivates BMP2 through CRE in the BMP2 promoter. (A) BMP2 promoter reporter activity in C2C12 cells, co-transfected with CREB plasmid and a series of truncated reporter constructs for 24 hours, was measured with β-gal normalization. (B) Sequence of −150/+165 of mouse BMP2 promoter. Putative sequences for CRE1, CRE2 and CRE3 are underlined and bolded. Sequences of ChIP primers are underlined only. (C) Sequences of mutant CRE1, CRE2 and CRE3 (mCRE1, mCRE2 and mCRE3). (D) BMP2 promoter reporter activity in C2C12 cells, co-transfected with wild-type −150/+165-Luc (WT) and mutant −150/+165-Luc in which CREs were mutated (mCRE1, mCRE2 and mCRE2), and with CREB plasmid for 24 hours, was measured. * p<0.01 (CREB transfection on mCRE2 vs WT or mCRE1 or mCRE3 reporters; mean±SE, n = 8). (E) Effects of PTH on BMP2 promoter activity. C2C12 cells were co-transfected with wild-type −150/+165-Luc (WT) and mutant −150/+165-Luc in which CRE2 was mutated (mCRE2) and treated with PTH at 50 nM for 24 hours. * p<0.01 (PTH mCRE2 vs PTH on WT reporter; mean±SE, n = 8).F) ChIP assay. Nuclear DNA-protein complexes were extracted from C2C12 cells treated with PTH at 50 nM for 6 hours and precipitated with anti-CREB antibody. PCR was performed to amplify the region of the BMP2 promoter that contained CRE2, using PCR primers indicated in (B). Input: BMP2 promoter DNA. IgG: Goat IgG as a negative control. (G) EMSA assay. Nuclear extracts of C2C12, 2T3 and MC3T3-E1 cells were incubated with a biotin-labeled DNA probe containing the CRE2 binding site sequence in the BMP2 promoter, in the absence or presence of anti-CREB antibody. The shift and super shift bands were analyzed using a 5% polyacrylamide gel.

CREB is known to transactivate target genes through a specific CRE consensus sequence in their promoters [17]–[22]. Sequence analysis revealed multiple CREs throughout the mouse BMP2 promoter from −2712 to +165 (data not shown). To identify the functional CRE(s) responsible for CREB transactivation of the BMP2 promoter, we first performed a promoter deletion study. C2C12 cells were transiently co-transfected with CREB expression plasmid or empty vector plus a series of truncated BMP2 promoter reporters representing −2712/+165, −2457/+165, −199/+165, −1803/+165, −968/+165, −838/+165, −310/+165, and −150/+165 of the 5′ promoter region of the BMP2 gene. Interestingly, we found that none of these progressively truncated BMP2 promoter reporters lost or reduced their responsiveness to CREB transactivation of luciferase activity (Fig. 5A). These results suggest that the potential CREs may be located within the −150/+165 region of the BMP2 promoter. Next, we analyzed the sequence of this narrow region in the promoter, and identified three putative CREs (CRE1, CRE2 and CRE3) in this area (Fig. 5B). To determine the role of these putative CREs in CREB transactivation, we performed site-directed mutagenesis. Each of these putative CREs located within the BMP2 promoter-luciferase construct (−150/+165-Luc) was mutated by deleting or replacing core nucleotides as shown in figure 5C. The results of luciferase reporter assays showed that levels of BMP2 transcriptional activity induced by CREB were comparable for mutant CRE1 and CRE3 reporters (mCRE1 and mCRE3) and the wild-type −150/+165-Luc reporter (Fig. 5D). Mutation of CRE2 (mCRE2), however, resulted in complete abrogation of the response to CREB stimulation (Fig. 5D), indicating that CRE2, in region −150/+165 of the BMP2 promoter, is a functional CRE responsible for CREB's action on the BMP2 gene. Furthermore, we also examined the effects of CRE2 mutation on PTH stimulation of BMP2 promoter activity. As shown in Figure 5E, we found that PTH lost its stimulatory effect on BMP2 promoter activity when the CRE2 is mutated, compared with wild-type CRE2 control.

We subsequently performed ChIP and EMSA assays to verify the interaction of CREB with CRE2 in the BMP2 promoter. In the ChIP assay, a 230 bp fragment of the BMP2 promoter containing the specific CRE2 sequence was amplified after the addition of anti-CREB antibody to nuclear DNA-protein complexes extracted from C2C12 cells, compared with non-specific IgG control (Fig. 5F, lane 3 vs lane 2). This suggests that CREB binds directly to CRE2 in the BMP2 promoter. Importantly, treatment of these cells with PTH (50 nM) for 6 hours further increased CREB binding ability to CRE2, evidenced by increased PCR product in the gel (Fig. 5F, lane 4 vs lane 3). Furthermore, EMSA was performed to obtain direct evidence of physical binding of CREB with CRE2. Nuclear proteins were extracted from C2C12, 2T3 and MC3T3-E1 osteoblast cells and reacted with a biotin-labeled DNA probe containing the CRE2 sequence (−13 to +20) of the BMP2 promoter. The addition of the CRE2 probe to these cell extracts induced a shift in mobility of the probe in the gel, (Fig. 5G, lane 2–4 vs lane 1), indicating a direct interaction between the CRE2 probe and nuclear proteins. Further, addition of anti-CREB antibody to the reaction mixture of C2C12 cells produced a super shift in mobility of the band (Fig. 5G, lane 7 vs lane 6), indicating that CREB binds directly to this CRE2 complex. Based on the combined results of luciferase reporter, ChIP and EMSA assays, we propose that the transcriptional factor CREB transactivates the BMP2 gene through a specific CRE2 site in the BMP2 promoter.

## Discussion

These collective results provide evidence that the PTH signaling pathway is an effective activator of BMP2 gene expression in osteoblasts, and this function is mediated by CREB transactivation of the BMP2 promoter. This transcriptional mechanism, at least in part, accounts for the anabolic function of the PTH-CREB pathway in osteoblast differentiation and bone formation.

It is well documented that intermittent dosing with PTH has an anabolic effect on bone [1]–[10], and that the PTH signaling pathway is a physiological regulator of osteoblast function in bone [11]–[30]. As a major mediator of PTH signaling, the transcriptional factor CREB has been found to play a role in osteoblast differentiation by regulating expression of osteoblast-specific genes [13]–[22], by which CREB may function as an anabolic regulator for bone mass. Several previous *in vivo* studies have provided evidence for the anabolic function of CREB in bone [30], [35], [36], [43], [44]. A recent report showed that small molecules with potent osteogenic activity induce osteoblast differentiation by activating intracellular CREB activity [43]. In studies with Lrp 5 knockout mice that exhibit low bone mass, Yadav et al reported that Lrp5 deficiency increases duodenal production of serotonin, which inhibits osteoblast function by suppressing CREB activity in osteoblasts [44]. Furthermore, they found that Lrp5 and CREB double knockout mice have a significantly lower bone mass than Lrp5 single null mutants [44]. Recently, we have reported that osteoblast-specific knockout of CREB in mice produces a skeletal phenotype with low bone mineral density and low bone volume [35], [36]. This is consistent with a previously reported similar osteopenic phenotype of the osteoblast-specific ICER (inducible cAMP early repressor) transgenic mice [30]. ICER belongs to the same bZIP family of transcription factors as CREB, and acts as a dominant negative regulator of gene transcription through CRE. Thus, it appears that CREB plays an important role in postnatal bone formation through its effect on osteoblast cells. To explore the mechanisms by which CREB exerts its effects on bone, in the current gain- and loss-of-function studies employing primary osteoblast cells and multiple osteoblast cell lines, we found that manipulation of activity and expression levels of PTH and CREB affects osteoblast function. We also demonstrated that the PTH-CREB signaling pathway is a positive regulator of osteoblast differentiation. These findings further support the hypothesis that promoting osteoblast differentiation is one of cellular mechanisms by which the PTH-CREB signaling pathway exerts its anabolic function in bone.

The cAMP-PKA-CREB axis represents a major signaling pathway that mediates PTH signaling [11]–[30]. In the current study, we demonstrated that PTH induces CREB phosphorylation in osteoblasts and that this function is regulated by pharmacological manipulation of the PTH signaling cascade in the upstream of CREB. The results of experiments with the cAMP-enhancer and the PKA-inhibitor suggest that the capacity of CREB to enhance BMP2 transcription in osteoblasts is phosphorylation-dependent. In these studies, however, we found that the PKA-inhibitor (KT5720) failed to completely abolish the capacity of CREB to increase BMP2 transcription in osteoblasts, suggesting that other protein kinases, in addition to PKA (such as protein kinase C), also may be involved in this process. Similar results have been reported with other cell systems [45], [46]. In a separate experiment, we found that the cAMP-enhancer IBMX had biphasic effects on the BMP2 promoter, depending on the duration of treatment. In contrast to the stimulatory effect on BMP2 expression observed in this study when cells were exposed to IBMX for only 24 hours, extending exposure up to 48 hours resulted in inhibition of BMP2 promoter activity (data not shown). This latter effect of IBMX on the BMP2 gene may be due to the degradation of transcription factor Gli2, which we previously identified as a stimulator of BMP2 expression in osteoblasts [39], [40].

CREB transactivates target genes through a consensus cAMP response element (CRE), by which CREB has been shown to transactivate multiple osteoblast maturation related marker genes, such as BSP and OCN [13]–[22]. The rationale in this study to focus on the BMP2 gene as a critical transcriptional target of CREB in osteoblasts includes the following: (1) BMP2 is the prototypic member of the BMP family responsible for osteoblast differentiation; (2) cAMP, an upstream activator of CREB, up-regulates BMP2 gene expression in other cells [37], and; (3) multiple putative CRE sites can be found throughout the BMP2 promoter. Therefore, we mapped out the molecular interaction between CREB protein and the BMP2 promoter. Utilizing promoter deletion, mutation, ChIP and EMSA studies, we defined a specific CRE (CRE2) located within the basal promoter loci from −150 to +165, as a functional binding element responsible for CREB transactivation of the BMP2 gene. We recognize, however, that regulation of the BMP2 gene is complex, and that multiple additional mechanisms, involving Gli proteins [39], [40], [47], β-catenin/TCF [48] and ERα [49], have been reported to contribute to the regulation of BMP2 transcription. Thus, we do not exclude the possibility that additional untested CRE sequences in the upstream of the BMP2 promoter may also possess potential cis-functions in BMP2 gene transcription in response to other nuclear transcription factors such as those identified above. Interestingly, in this study, we found that ATF4 (a member of the CREB/ATF family) failed to stimulate BMP2 expression. In other cell types, ATF4 and CREB have been shown to share the same DNA binding element (TGACGTCA) in transactivating the promoter activities of target genes [49]. We presume, therefore, that CREB transactivation of the BMP2 gene in osteoblasts may require other co-activators associated within the CREB transcriptional machinery that are structurally and functionally distinct from those associated with ATF4 action. However, more comparison studies about the transcriptional mechanisms of these factors on the BMP2 gene are needed to examine this possibility.

The experiments in which noggin blocked CREB-induced ALP activity support the concept that the function of CREB in osteoblasts is likely BMP-dependent, because noggin blocks BMP signaling by hindering BMP ligands from binding to BMP receptors [50], [51]. The present data showed that CREB, as a powerful stimulator of BMP2 transcription, lacks the capability to induce expression of BMP4, the closest member of BMP2 in the BMP family. However, it is possible that CREB may have a positive regulating function for transcription of other BMP family members. This possibility warrants further investigation.

In addition to the capability of increasing BMP2 gene expression in osteoblasts demonstrated in the current study, the PTH-cAMP-CREB pathway also has been shown to enhance BMP2 activity. For example, it has been demonstrated that this pathway is capable of enhancing BMP2-stimulated Smad signaling [34], and synergizing the BMP2-induced anabolic effects on differentiation of osteoblasts [23], [25], [26] and chondrocytes [23], [52] *in vitro*, and new bone formation *in vivo* [28], [29]. In contrast, BMP2 is also known to have a positive feedback effect on CREB function. In osteoblast precursor C2C12 cells, BMP2 synergizes with PKA-CREB to enhance ALP activity [25]. Therefore, the crosstalk between the PTH-CREB and BMP2 signaling pathways is complex, and could occur at multiple steps in these signaling cascades. In order to further test the role of BMP2 in mediating the function of PTH-CREB signaling in osteoblast differentiation and bone formation, a BMP2 invalidated mouse model is needed. We are now proposing a study using osteoblast-specific BMP2 knockout mice to determine the effects of BMP2 deficiency on the anabolic effects of intermittent PTH on bone mass. The present finding that PTH-CREB up-regulates of BMP2 gene expression in osteoblasts provides new insights into the relation between these important anabolic signaling pathways in bone.

In summary, the functional and mechanistic studies in this study demonstrate that PTH signaling is a positive regulator of BMP2 gene expression in osteoblasts, mediated directly by CREB transactivation of BMP2 promoter activity. This transcriptional mechanism contributes to osteoblast differentiation. The collective results of these studies support the hypothesis that the anabolic effects of PTH on bone mass are mediated through the cAMP-PKA-CREB-BMP2 pathways.

## Materials and Methods

### DNA constructs and compounds

The CREB expression plasmid pRC/RSV-CREB, its empty vector pRC/RSV, and CREB responsive reporter (CRE-Luc) containing multiple CREs were obtained from Dr. Richard Goodman [53]. The ATF4 expression vector was a gift of Dr. Xiangli Yang [42]. Mouse BMP2 promoter luciferase reporter (−2712/+165-Luc) was constructed by linking the 5′ promoter region (−2712/+165) of mouse the BMP2 gene to firefly luciferase in a pGL3 vector [39]–[40]. The promoter was truncated from the distal end by enzymatic digestion to create a series of deletion constructs, including −2457/+165-Luc, −199/+165-Luc, −1803/+165-Luc, −968/+165-Luc, −838/+165-Luc, −310/+165-Luc, and −150/+165-Luc. Mouse BMP4 promoter reporter (BMP4-Luc) was obtained from Dr. Stephen Harris. The CREB siRNA (CREB ShortCut® siRNA Mix), and negative control siRNA (New England Biolabs, Beverly, MA), IBMX and KT5720 (Calbiochem, San Diego, CA), and PTH and noggin (R&D Systems, Minneapolis, MN), was purchased.

### Culture of primary osteoblast cells and osteoblast cell lines

Osteoblastic cell lines: C2C12 (ATCC, #CRL-1772), 2T3 [54], UMR106 (ATCC, #1661), and MC3T3-E1 (ATCC, #2593) cells were seeded into 6 to 48-well plates with complete culture media (DMEM for C2C12 and UMR106 cells and αMEM for 2T3 and MC3T3-E1 cells), supplemented with 10% fetal calf serum (FCS), 1% penicillin/streptomycin and 1% L-Glutamine, at a cell density that allowed cells to reach 60–70% confluence after overnight culture in 5% CO_2_ at 37°C. Calvarial osteoblastic cells: Calvarial bones were dissected from newborn osteoblast-specific CREB knockout mice and their control littermates. CREB knockout mice, which exhibit low bone mass [35], [36], were generated by crossing CREBfloxed mice [55] with type I collagen I-á1 Cre (Col1a1-2.3-Cre) mice. These mice were housed following standard LAR mouse housing protocol. All the mouse manipulations were performed under a Tulane IACUC approved protocol (#4196). Calvarial tissues were digested with 0.05% trypsin and 1.5 U/ml collagenase at 37°C on a rocking platform. Cell fractions 2 to 4 were collected and cultured in αMEM.

### CREB phosphorylation assay

C2C12 cells cultured in 6-well plates were incubated with PTH, IBMX or KT5720 at different doses for 0 to 4 hours. Cells were lysed with SDS-RIPA buffer containing protease inhibitors PMSF (1 mM), aprotinin (10 µg/ml) and leupeptin (10 µg/ml). Samples were loaded on SDS-PAGE (Mini-Protein II Ready Gels, Bio-Rad, Hercules, CA), and proteins were electrophoretically separated under reducing conditions and then transferred onto PVDF membranes (Bio-Rad) in transblotting buffer (25 mM Tris, 192 mM glycine and 20% [v/v] methanol, pH 8.3) at 4°C for 1 hour. After blocking with 5% milk in TBS-T (0.1% Tween 20) for 1 hour at room temperature, membranes were incubated with antibodies against phosphorylated-CREB (Phospho-CREB, Ser133) or non-phosphorylated CREB (Cell Signaling Technology Inc. Danvers, MA) in 5% milk in TBS-T at 4°C overnight. After washes, membranes were incubated with horse-radish peroxidase (HRP)-conjugated secondary antibody (Amersham Biosciences, Buckinghamshire, UK) at 1∶5,000 dilution at room temperature for 1 hour, then washed 6 times with TBS-T buffer for 5 minutes each. Immunoblots were detected using an enhanced chemiluminesence ECL system (Amersham). The antibodies on the same PVDF membranes were removed with stripping buffer and re-blotted with anti-β-actin antibody to detect β-actin levels for protein normalization.

### Alkaline phosphatase (ALP) activity assay

All the tested osteoblast cells were seeded into 48-well plates. To test the effects of PTH, C2C12 cells were incubated with PTH at different doses for 24 hours. To test the effects of CREB overexpression, C2C12 and 2T3 cells were transiently transfected with CREB expression vector or empty vector using LipofectAmine Plus reagent (Invitrogen, Carlsbad, CA) following the manufacturer's protocol. The transfected cells were cultured in the presence or absence of noggin at 500 ng/ml for 48 hours. To test the effects of CREB knockout, primary calvarial cells were harvested and cultured for 24 hours. All these cells were cultured in media supplemented with 2.5% FCS. Then, cells were lysed in 0.05% Triton X-100 buffer. ALP activity in cell lysates was spectrophotometrically quantitated at 405 nm using Sigma ALP reagents (Sigma-Aldrich, St. Louis, MO) and normalized with total cellular protein.

### Mineralized matrix formation

Osteoblastic 2T3 cells were transfected with CREB expression vector or empty vector in 24-well plates and cultured with αMEM with 10% FCS. At confluence, the culture medium was supplemented with 5% FCS, ascorbic acid (100 µg/ml), and β-glycerol phosphate (5 mM). The medium was refreshed every other day. At days 14 and 21, the cultures were terminated and fixed in phosphate-buffered formalin for 10 minutes followed by von Kossa staining with 2% silver nitrate solution, and exposed to sunlight for 20 minutes. The area of mineralized matrix was stained black in the wells.

### Real time PCR

Total RNA was purified from osteoblast cells in 6-well plates with various treatments, and reverse transcribed into cDNA. Quantitative PCR of mouse BMP2 mRNA was performed using a cDNA template and mouse BMP2 primers/probe, Mm01340178_m1 (Applied Biosystems, Carlsbad, CA) on 7300 Real Time PCR System (Applied Biosystems). The endogenous control was GAPDH detected using a VIC/MGB Probe (4352339E, Applied Biosystems). mRNA levels of other osteoblast marker genes including Runx2, Col1a1 and OCN were also quantitated by real time PCR using primers Mm00501578_m1, Mm00801666_g1*, Mm03413826_mH (Applied Biosystems) respectively, with 18s rRNA for normalization.

### Promoter reporter assay

C2C12 cells in 24-well plates were transfected with 0.5 µg of CREB responsive reporter CRE-Luc, or mouse BMP2 promoter luciferase reporter −2712/+165-Luc, or its truncated forms, or mouse BMP4 promoter reporter BMP4-Luc, and 0.1 µg of pSV-β-Galactosidase (β-Gal) expression vector (Promega, Madison, WI), using the LipofectAmine Plus Reagent (Invitrogen). Cells transfected with the BMP2 or BMP4 promoter reporters were also co-transfected with 0.2 µg of CREB or ATF4 expression constructs. The cells were cultured in the presence or absence of experimental compounds including PTH, IBMX or KT5720 for 24–48 hours and then lysed with Reporter Lysis Buffer (Promega). Luciferase activities of cell lysates were measured by a luciferase reporter assay kit (Promega) and normalized to β-Gal activity.

### RNA interference

C2C12, 2T3 and UMR106 cells cultured in 6-well plates were transiently transfected with CREB siRNA or negative control siRNA (CREB ShortCut® siRNA Mix, New England Biolabs) using siRNA transfection reagents. Following confirmation of CREB protein levels in the cell lysates by Western blot at 24 to 48 hours after transfection (data not shown), BMP2 mRNA levels were determined as described above.

### Mutation of promoter binding sites

The putative CREs in the BMP2 promoter reporter construct −150/+165-Luc were mutated by deleting or replacing core nucleotides using GeneEditor™ in Vitro Site-Directed Mutagenesis System (Promega) following the manufacturer's protocol. The phosphorylated mutant primers for individual mutation of CRE1, CRE2 and CRE3 were p-tcgcggcccagctagcagaacgtccgtc, p-ctggtccctgaggccggatccagc agcagccttgcc, and p-cctgctcgaggctgtggatccagcacttggctggag, respectively. The luciferase activity of mutant reporters responding to CREB transfection were measured as described above.

### Chromatin immunoprecipitation (ChIP)

C2C12 cells cultured in 100 mm Petri dishes were incubated with PTH at 50 nM for 6 hours. The cells were fixed with 1% formaldehyde for 10 minutes to cross-link the chromatin. The ChIP assay was performed following the protocol of the ChIP kit (Upstate, MA). Briefly, cells were scraped and sonicated on ice to shear chromatin DNA down to 0.2–1.0 kb fragments. The sonicated cell supernatants were pre-cleared with a Protein A agarose/salmon sperm DNA slurry. Anti-CREB antibody (Cell Signaling Technology, Inc.) was added to the supernatant at 4°C overnight, followed by incubation with fresh Protein A agarose beads for 1 hour at 4°C for precipitation. The specific protein-DNA complex was disassociated and DNA fragments were purified. With these DNA fragments as templates, PCR was performed using primers F-5′ggcatcgcccacgatggctg and R-5′caagttcaagaagtctcc, to amplify a sequence containing a specific CRE in the BMP2 promoter. The BMP2 promoter DNA input was used as a positive control. Goat IgG served as a negative control.

### Electrophoresis mobility shift assay (EMSA)

C2C12, 2T3, and MC3T3-E1 cells (approximate 2–4×10^6^) were collected by digestion, and the nuclear extracts were prepared using a kit (Pierce). A synthesized, biotin-labeled and annealed DNA probe (5′tggtccctgaggccgacgacagcagcagccttgc) at 20 fmols, which contained the CRE2 binding site sequence in the BMP2 promoter, was reacted with nuclear extracts for 20 minutes. The reactions were applied onto 5% polyacrylamide gels (Bio-Rad), and then transferred to Nylon membranes. Biotin-labeled DNA was detected using a streptavidin-horseradish peroxidase conjugate and chemiluminescent substrate (Pierce). To examine super shift of DNA-protein complexes, an antibody against CREB was added to DNA-protein binding reactions and loaded to the gels. The detailed methods followed the protocol of LightShift Chemiluminescent EMSA Kit (Pierce).

### Statistical analysis

Student's two-tailed unpaired *t* test was used to examine the significance changes in these experiments.

## Supporting Information

Figure S1 **Quantitation of phosphor-CREB.** Cq-Fq: Quantitation of phosphor-CREB. C2C12 cells were treated with IBMX at 0 to 300 µM for 1 hour (Cq); IBMX at 50 µM for 0 to 4 hours (Dq); or KT5720 at 0 to 10 µM for 1 hour (Eq); KT5720 at 10 µM for 0 to 2 hours (Fq). Phospho-CREB was detected by Western blot. The signal intensity of Western blot band was quantitated using GelScan v 5.1 (BioSciTech) with normalization by the intensity of β-actin.
(TIF)
