# Supplementary figures and images for: Transcriptional Regulation of BMP2 Expression by the PTH-CREB Signaling Pathway in Osteoblasts
Source: PLoS One. 2011 Jun 9;6(6):e20780. doi: 10.1371/journal.pone.0020780 (PMC3111437; doi:10.1371/journal.pone.0020780)

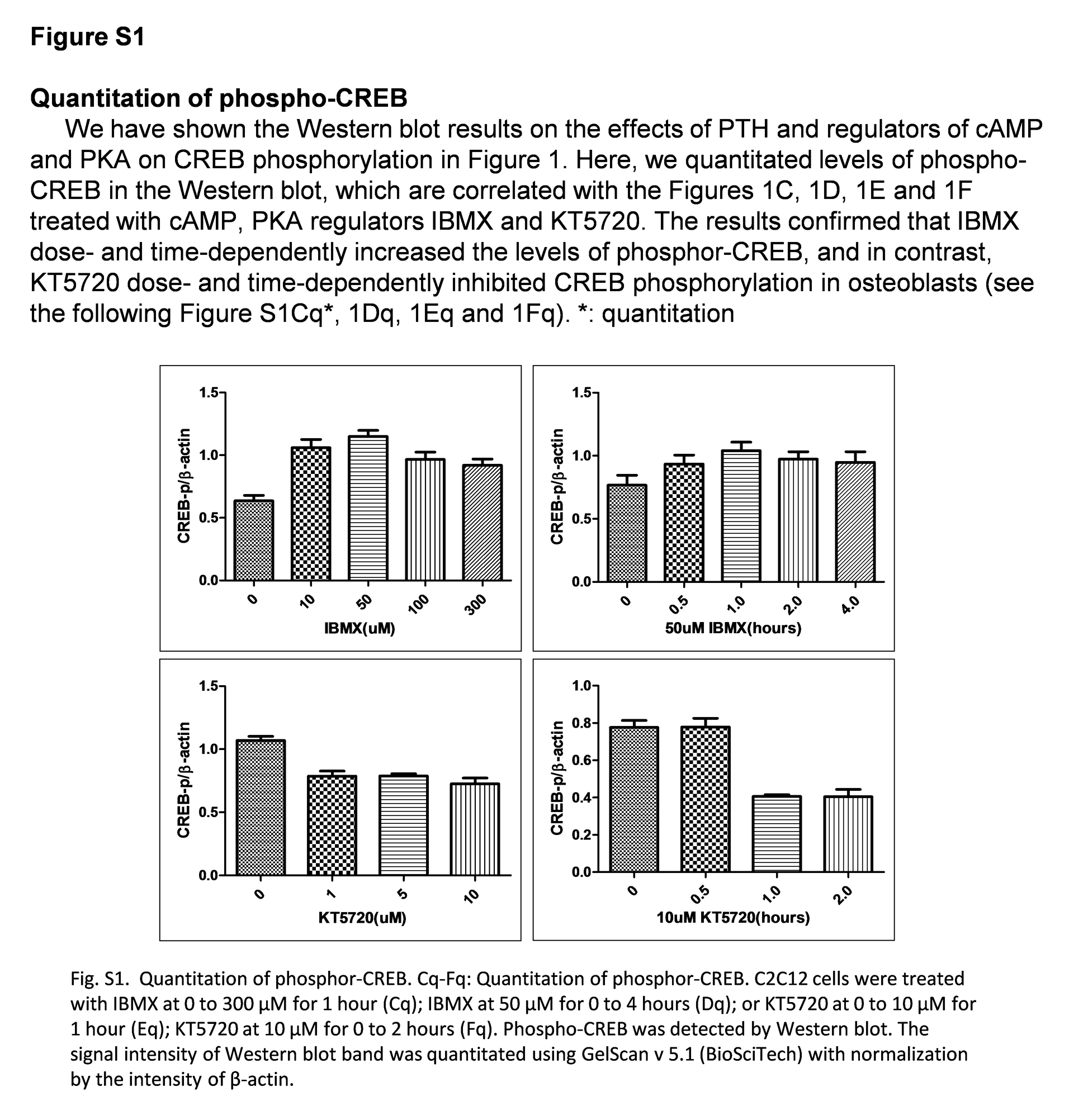

Supplement: Figure S1 — Quantitation of phosphor-CREB. Cq-Fq: Quantitation of phosphor-CREB. C2C12 cells were treated with IBMX at 0 to 300 µM for 1 hour (Cq); IBMX at 50 µM for 0 to 4 hours (Dq); or KT5720 at 0 to 10 µM for 1 hour (Eq); KT5720 at 10 µM for 0 to 2 hours (Fq). Phospho-CREB was detected by Western blot. The signal intensity of Western blot band was quantitated using GelScan v 5.1 (BioSciTech) with normalization by the intensity of β-actin. (TIF) [file pone.0020780.s001.tif]
